# Deconditioning does not explain orthostatic intolerance in ME/CFS (myalgic encephalomyelitis/chronic fatigue syndrome)

**DOI:** 10.1186/s12967-021-02819-0

**Published:** 2021-05-04

**Authors:** C. (Linda) M. C. van Campen, Peter C. Rowe, Frans C. Visser

**Affiliations:** 1 Stichting CardioZorg, Planetenweg 5, 2132 HN Hoofddorp, Netherlands; 2 Department of Paediatrics, Johns Hopkins University School of Medicine, Baltimore, MD USA

**Keywords:** Chronic fatigue syndrome, Myalgic encephalomyelitis, Peak oxygen consumption, Cardiopulmonary exercise test, Deconditioning, Cerebral blood flow, POTS, Orthostatic hypotension, Orthostatic intolerance, Head-up tilt testing

## Abstract

**Background:**

Orthostatic intolerance (OI) is a frequent finding in individuals with myalgic encephalomyelitis /chronic fatigue syndrome (ME/CFS). Published studies have proposed that deconditioning is an important pathophysiological mechanism in various forms of OI, including postural orthostatic tachycardia syndrome (POTS), however conflicting opinions exist. Deconditioning can be classified objectively using the predicted peak oxygen consumption (VO_2_) values from cardiopulmonary exercise testing (CPET). Therefore, if deconditioning is an important contributor to OI symptomatology, one would expect a relation between the degree of reduction in peak VO_2_during CPET and the degree of reduction in CBF during head-up tilt testing (HUT).

**Methods and results:**

In 22 healthy controls and 199 ME/CFS patients were included. Deconditioning was classified by the CPET response as follows: %peak VO_2_ ≥ 85% = no deconditioning, %peak VO_2_ 65–85% = mild deconditioning, and %peak VO_2_ < 65% = severe deconditioning. HC had higher oxygen consumption at the ventilatory threshold and at peak exercise as compared to ME/CFS patients (p ranging between 0.001 and < 0.0001). Although ME/CFS patients had significantly greater CBF reduction than HC (p < 0.0001), there were no differences in CBF reduction among ME/CFS patients with no, mild, or severe deconditioning. We classified the hemodynamic response to HUT into three categories: those with a normal heart rate and blood pressure response, postural orthostatic tachycardia syndrome, or orthostatic hypotension. No difference in the degree of CBF reduction was shown in those three groups.

**Conclusion:**

This study shows that in ME/CFS patients orthostatic intolerance is not caused by deconditioning as defined on cardiopulmonary exercise testing. An abnormal high decline in cerebral blood flow during orthostatic stress was present in all ME/CFS patients regardless of their %peak VO_2_ results on cardiopulmonary exercise testing.

**Supplementary Information:**

The online version contains supplementary material available at 10.1186/s12967-021-02819-0.

## Introduction

Orthostatic intolerance (OI) is a frequent finding in individuals with myalgic encephalomyelitis /chronic fatigue syndrome (ME/CFS). The prevalence of OI symptoms in adults with ME/CFS varies between 28 and 96% in published studies [1]. This large variation is due to differences in patient selection, methodology of orthostatic testing and the comprehensiveness of ascertainment of orthostatic symptoms. In recent studies we found a prevalence of OI symptoms of 82% in adults and 96% in adolescents [2, 3]. OI is now considered one of the cardinal features of ME/CFS [1].

Some authors have proposed that deconditioning is an important pathophysiological mechanism in various forms of OI, including postural orthostatic tachycardia syndrome (POTS) [4–10]. In contrast, the 2015 Heart Rhythm Society Expert Consensus document on POTS states: “it is unclear whether deconditioning is the primary cause or a secondary phenomenon” [11].

Parsaik et al. suggested that the degree of deconditioning can be demonstrated by cardiopulmonary exercise testing (CPET) in patients with OI. The authors defined a percentage peak oxygen consumption (VO_2_) cut-off value of ≥ 85% as indicating the absence of deconditioning, 65–84% as mild deconditioning, and < 65% as severe deconditioning [6].

We recently reported that extracranial Doppler imaging of the internal carotid and vertebral arteries can measure the total cerebral blood flow (CBF) during head-up tilt testing (HUT), and thereby provides an objective confirmation of OI. In healthy adults, this technique identified a mean 7% reduction in CBF during a 30 min HUT. In ME/CFS patients however, the mean reduction in CBF was 26% [3]. Moreover, there was a significant relation between the CBF reduction and OI symptomatology during the tilt test. These data suggest that CBF measurements are more sensitive for OI in ME/CFS patients during HUT than the standard methods of classifying OI based on heart rates and blood pressures alone.

If deconditioning is an important contributor to OI symptomatology, then a relation between the degree of reduction in peak VO_2_ during CPET and the reduction in CBF during HUT is to be expected. To investigate this hypothesis we examined all ME/CFS patients who had undergone both a CPET and CBF measurements during HUT.

## Patients, material and methods

This was a retrospective study of patients referred between October 2012 and August 2020 to the Stichting CardioZorg, a cardiology clinic that specializes in the assessment and treatment of those with CFS and ME. All eligible participants had been referred by their general practitioners for the diagnosis of ME/CFS. Patients underwent a detailed clinical history, physical examination, laboratory analysis, ECG and echocardiography. Based on their symptoms, we established the diagnosis of chronic fatigue syndrome (CFS) according to the Fukuda Criteria [12] and myalgic encephalomyelitis (ME) according to the international ME criteria [13]. In all patients, alternative diagnoses which could explain the fatigue and other symptoms were ruled out.

From the entire referred population, patients were selected for this study if the HUT and a CPET were available and performed within a 1 year interval. HUT was performed because of the clinical suspicion of OI. CPET was performed for a variety of reasons: assessment of the heart rate (HR) at the ventilatory threshold (VT), to guide exercise activity [14, 15], to demonstrate reduction of the exercise capacity on day two of a 2-day CPET protocol [16–18], and to assess the degree of disability for social security claims.

This time interval of 1 year was intended to ensure that the clinical condition of the patient was relatively stable. Additionally, ME/CFS patients were selected with a proven significant CBF reduction (see below). For comparison, healthy controls (HC) who also underwent HUT and CPET within an interval of 3 month, were included. We excluded patients with a body mass index (BMI) of ≥ 37 [19], with an insufficient quality of the Doppler investigation, and if patients were using medications that lowered heart rate or blood pressure.

The changes in HR and blood pressure (BP) during head-up tilt test were classified according to the consensus statements [11, 20, 21]: normal HR and BP response, classic orthostatic hypotension (cOH; a decrease of over 20 mmHg in systolic blood pressure (SBP) and over 30 mmHg in the event of a SBP over 160 mmHg [22], or a decrease of 10 mmHg in diastolic blood pressure (DBP) from 1 to 3 min after onset of the tilt), delayed orthostatic hypotension (dOH; similar changes in BP as for classic orthostatic hypotension but developing after 3 min after onset of tilt), POTS (a sustained increase of at least 30 bpm within 10 min of tilting, without a significant decrease in BP), and syncope or near-syncope (VVS).

The study was carried out in accordance with the Declaration of Helsinki. All ME/CFS participants and HC gave informed, written consent authorizing us to use their medical records for research purposes. The study of the use of clinical data was approved by the medical ethics committee of the Slotervaart Hospital, number P1736, Amsterdam, NL. The testing of HC was approved by the same ethics committee, number P1411.

### Head-up tilt test with cerebral blood flow measurements

Measurements were performed as described previously [23]. Briefly, all participants were positioned for 20 min in a supine position before being tilted head-up to 70 degrees for a maximum of 30 min. HR, SBP and DBP were continuously recorded by finger plethysmography using the Nexfin device (BMeye, Amsterdam, NL) [24, 25]. After the test, HR and BP’s were extracted from the Nexfin device and imported into an Excel spreadsheet. Internal carotid artery (ICA) and vertebral artery (VA) Doppler flow velocity frames were acquired by one operator in the supine position and twice during the upright phase, using a Vivid-I system (GE Healthcare, Hoevelaken, NL) equipped with a 6–13 MHz linear transducer. High resolution B mode images, color Doppler images, and the Doppler velocity spectrum (pulsed wave mode) were recorded in one frame. At least two consecutive series of six cardiac cycles per artery were recorded.

Calculations of blood flow were performed as described previously [23] by another operator, unaware of the clinical data. In one cardiac cycle CBF was calculated from the mean blood flow velocity x the mean surface area. To compensate for respiratory variation, flow in the four arteries was calculated in 6 cardiac cycles and data were averaged. Total cerebral blood flow was calculated by adding the flow of the four arteries. For the present study the supine CBF and the CBF at the end of the upright phase of the HUT were taken. The end tilt CBF measurement was expressed as the percent reduction compared to the supine CBF. Because CBF Doppler measurements required approximately 3 min, we excluded those with rapid development of cOH and VVS because it was impossible to completely assess the 4 cerebral arteries while blood pressure was dropping rapidly. As cerebral blood flow is age dependent, differences between supine and end-tilt are shown as a percent reduction to enable comparison of ME/CFS patients with a broad age range [26–29].

Based on our previous study, we considered OI to be confirmed by CBF measurements if a reduction greater than 2 SD beyond the mean of the healthy volunteers [30]. This defines an abnormal CBF result as a > 13% reduction during tilt.

### Cardiopulmonary exercise testing

Patients underwent a symptom-limited exercise test on a cycle ergometer (Excalibur, Lode, Groningen, The Netherlands) according to a previously described protocol [31]. Briefly, a ramp workload protocol was used, varying between 10–30 W/min. Oxygen consumption (VO_2_ in ml/min/kg), carbon dioxide release (VCO_2_ in ml/min/kg), and oxygen saturation were continuously measured (Cortex, Procare, The Netherlands), and displayed on screen using Metasoft software (Cortex, Biophysic Gmbh, Germany). An ECG was continuously recorded and HR and BP were measured using the Nexfin device (BMEYE, Amsterdam, The Netherlands) [25]. The metabolic measurement system (Cortex, Biophysic Gmbh, Germany) was calibrated before each test with ambient air, standard gases of known concentrations, and a 3-L calibration syringe. The VT, a measure of the anaerobic threshold, was identified from expired gases using the V-Slope algorithm [32]. An experienced cardiologist supervised the test and performed visual assessment and confirmation of the algorithm-derived VT. The peak VO_2_ was defined as the mean of the VO_2_ measurements of the last 15 s before ending the exercise. VO_2_ at the VT and peak were expressed as a percentage of the normal values of a population study: %VT VO_2_, %peak VO_2_ [19]. Also, the mean respiratory exchange ratio (RER; VCO_2_/VO_2_) of the last 15 s was calculated [33]. As absolute oxygen consumption differs between males and females and are age related results are shown in percent of a reference group to enable comparison of both genders and a broad age range [34–39].

### Statistical analysis

Data were analyzed using Graphpad Prism version 8.2.4 (Graphpad software, La Jolla, California, USA). All continuous data were tested for normal distribution using the D’Agostino & Pearson omnibus normality test, and presented as mean (SD) or as median (IQR), where appropriate. Nominal data (gender, hemodynamic tilt test results, oxygen consumption: normal, moderate deconditioning, and severe deconditioning) were compared using the Chi-square test (up to a 3 × 3 table). For continuous data, groups were compared using the paired or unpaired t-test or with a Wilcoxon matched-pairs signed rank test or Mann–Whitney test where appropriate. Within group comparison was performed using the ordinary one-way analysis of variance (ANOVA) or Kruskal–Wallis test where appropriate. Where significant, results were then explored further using the post-hoc Tukey’s test or Dunn’s test where appropriate. Within-group comparison was performed using the two-way ANOVA. Where significant, results were then explored further using the post-hoc Holm-Sidak test. Linear regression was performed to assess the relation between measures (percent CBF reduction from supine to end-tilt and percent peak VO_2_ for all patients shown for different hemodynamic results on HUT as POTS etc., HC as a group and results split in time intervals). We elected to use a more conservative p-value of < 0.01 to indicate statistical significance.

## Results

### Participants

Between October 2012 and August 2020, 1124 patients met the criteria for ME/CFS. In 916 patients a HUT was performed because of the clinical suspicion of OI. Of those 916 patients 65 patients were excluded because of insufficient quality or missing data of the CBF measurements, the presence of cOH and VVS, or the use of HR and BP lowering drugs. Of the remaining 851 patients, 249 underwent at least one cardiopulmonary exercise test. Of those 249, two were excluded due to HR and BP lowering medication use, none because of a BMI ≥ 37. In 199 patients the interval between the HUT and CPET was less than 1 year (mean interval 4 ± 3 month) and in 48 patients the interval was more than 1 year. Review of the patient charts showed that none of the 199 patients had a major change in symptoms or function due to an adverse event like surgery, trauma, or a serious infection like EBV. Twenty-eight subjects of the group with a normal HR and BP (normHRBP) response during HUT (21%) showed a normal CBF reduction, while the remaining 105 (79%) showed an abnormal CBF reduction. All patients with POTS (n = 39) and dOH (n = 27) showed an abnormal CBF reduction, leading to a total of 171 patients to be studied. In 67 patients, HUT was performed after the CPET, in 106 patients HUT was performed before the CPET. There were no differences in demographic data between ME/CFS patients who were included or excluded from the study (data not shown). Twenty-two HC fulfilled the inclusion criteria of undergoing a HUT and CPET within the fixed study interval of one year and had a normal HR and BP response during HUT. None of them used medication except for the occasional use of pain medication. The data of HC and patients with a normal HR and BP response and a normal CBF reduction are provided in Additional file 1 data section.

Table 1 shows the demographic data of the ME/CFS patients with a normal HR and BP response (n = 105), POTS (n = 39), and dOH (n = 27). Demographics did not differ significantly between the three groups.

**Table 1:** Demographic data of ME/CFS patients with a normal HR and BP response (group 1), ME/CFS patients with POTS (group 2), and ME/CFS patients with dOH (group as observed during HUT), all with a significant CBF decrease as measured during HUT

	Group 1 NormHRBP (n = 105)	Group 2 POTS (n = 39)	Group 3 dOH (n = 27)	Ordinary one-way ANOVA with post hoc Tukey’s test
Male/female	21/84	10/29	5/22	Chi-square test: p = 0.72 (2 × 4 table)
Age (years)	41 (9)	38 (11)	43 (12)	F (2, 168) = 1.99; p = 0.14
Height (cm)	171 (8)	175 (10)	173 (9)	F (2, 168) = 2.39; p = 0.09
Weight (kg)	73 (16)	75 (14)	73 (14)	F (2, 168) = 0.31; p = 0.73
BMI (kg/m^2^)	24.9 (4.8)	24.6 (3.8)	24.5 (5.3)	F (2, 168) = 0.08; p = 0.92
BSA (m^2^)	1.85 (0.20)	1.90 (0.21)	1.86 (0.17)	F (2, 168) = 1.02; p = 0.36
Disease duration (yrs)	11 (5–16.5)	7 (4–13)	11 (4–18)	Kruskal–Wallis test: X2 = 4.21; p = 0.12

*BMI* body mass index, *BSA* body surface area (formula DuBois), *Dis duration* disease duration, *NormHRBP* normal heart rate and blood pressure response during HUT, *dOH* delayed orthostatic hypotension during HUT, *POTS* postural orthostatic tachycardia syndrome during HUT, *yrs* years given as median with IQR

Table 2 shows the results of the CPET in the three ME/CFS groups. None of the studied parameters differed between the three groups. Table 3 shows the hemodynamic and CBF data during HUT of the three patient groups. By definition, end-tilt HR was highest in the POTS patient group compared the other two patient groups (both p < 0.0001). By definition, end-tilt SBP in patients with dOH was lower compared to the other two patient groups but only reached significance in the comparison of dOH patients vs the normHRBP patients: p < 0.0001. Similarly, end-tilt DBP was lower in the dOH patients compared to the other two patient groups, but only reached significance in the comparison of dOH patients vs the normHRBP patients: p < 0.0001. Baseline, end tilt CBF, and the %CBF reduction were not different between the three patients groups.

**Table 2:** CPET data in ME/CFS patients with a normal HR/BP response (group 1), ME/CFS patients with POTS (group 2), and ME/CFS patients with dOH (group 3), all with a significant CBF decrease as measured during HUT

CPET data	Group 1 NormHRBP (n = 105)	Group 2 POTS (n = 39)	Group 3 dOH (n = 27)	Ordinary one-way ANOVA with post hoc Tukey’s test
HR rest (bpm)	86 (14)	86 (12)	86 (15)	F (2, 168) = 0.02; p = 0.98
HR peak (bpm)	147 (23)	146 (19)	146 (25)	F (2, 168) = 0.03; p = 0.97
SBP rest (mmHg)	125 (16)	120 (14)	128 (15)	F (2, 168) = 1.25; p = 0.29
DBP rest (mmHg)	84 (10)	81 (11)	83 (12)	F (2, 168) = 0.67; p = 0.52
SBP peak (mmHg)	165 (26)	158 (26)	161 (26)	F (2, 168) = 0.79; p = 0.46
DBP peak (mmHg)	99 (13)	92 (13)	96 (12)	F (2, 168) = 2.36; p = 0.10
VT VO_2_ (ml/min/kg)	12 (4)	11 (3)	12 (4)	F (2, 168) = 1.28; p = 0.28
Peak VO_2_ (ml/min/kg)	21 (7)	19 (5)	21 (7)	F (2, 168) = 0.80; p = 0.45
%VT VO_2_	41 (11)	36 (11)	41 (12)	F (2, 168) = 2.81; p = 0.06
%peak VO_2_	70 (21)	62 (16)	71 (21)	F (2, 168) = 2.59; p = 0.08
RER	1.06 (0.12)	1.11 (0.12)	1.09 (0.12)	F (2, 168) = 2.12; p = 0.12

*DBP* diastolic blood pressure, *dOH* delayed orthostatic hypotension during HUT, *HR* heart rate, *NormHRBP* normal heart rate and blood pressure response during HUT, *POTS* postural orthostatic tachycardia syndrome during HUT, *RER* respiratory exchange ratio, *SBP* systolic blood pressure, *VO2* oxygen consumption in ml/min/kg, *VT* ventilatory threshold, *% VO*_*2*_ oxygen consumption as percentage of normal values of a population study (19)

^#^A p-value of < 0.01 was considered significantly different for this study

**Table 3:** Hemodynamic data during HUT in ME/CFS patients with a normal HRBP response (group 1), with POTS (group 2) and with dOH (group 3), all with a significant CBF decrease as measured during HUT

HUT data	Group 1 NormHRBP (n = 105)	Group 2 POTS (n = 39)	Group 3 dOH (n = 27)	Ordinary one-way ANOVA with post hoc Tukey’s test
HR supine (bpm)	73 (11)	77 (14)	73 (10)	F (2, 168) = 1.83; p = 0.16
HR end tilt (bpm)	90 (14)	114 (24)	90 (16)	F (2, 168) = 29.52; p < 0.0001; 1vs2 p < 0.0001; 1vs3 p = 0.99; 2vs3 p < 0.0001
SBP supine (mmHg)	134 (15)	132 (16)	142 (19)	F (2, 168) = 3.14; p = 0.04#
SBP end tilt (mmHg)	131 (16)	119 (24)	109 (16)	F (2, 168) = 19.14; p < 0.0001; 1vs2 p = 0.0008; 1vs3 p < 0.0001; 2vs3 p = 0.09
DBP supine (mmHg)	79 (8)	78 (8)	81 (9)	F (2, 168) = 1.21; p = 0.30
DBP end tilt (mmHg)	84 (9)	81 (16)	72 (13)	F (2, 168) = 10.91; p < 0.0001; 1vs2 p = 0.25; 1vs3 p < 0.0001; 2vs3 p = 0.01#
CBF supine (ml/min)	628 (107)	608 (96)	611 (98)	F (2, 168) = 0.70; p = 0.50
CBF end tilt (ml/min)	456 (83)	435 (79)	435 (79)	F (2, 168) = 1.29; p = 0.28
%CBF reduction	− 27.4 (6.1)	− 28.6 (5.3)	− 28.8 (4.9)	F (2, 168) = 0.98; p = 0.38

*CBF* cerebral blood flow, *DBP* diastolic blood pressure, *dOH* delayed orthostatic hypotension during HUT, *HR* heart rate, *HUT* head-up tilt test, *NormHRBP* normal heart rate and blood pressure response during HUT, *POTS* postural orthostatic tachycardia syndrome during HUT, *SBP* systolic blood pressure

^#^A p-value of < 0.01 was considered significantly different for this study

**Fig. 1:**
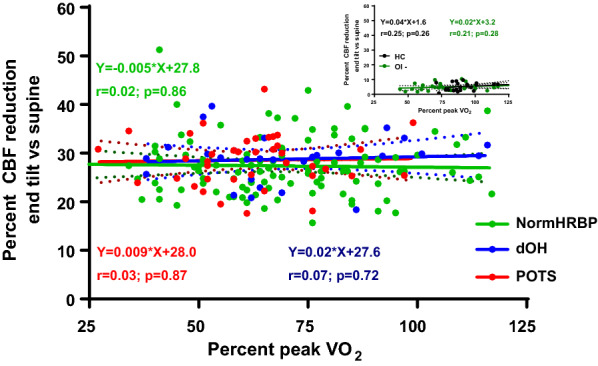
Correlation between the percent CBF reduction at end-tilt compared to supine and the percentage peak VO_2_ for all ME/CFS patients (n = 199 and HC (n = 22). *CBF* cerebral blood flow in ml/min/kg, *dOH* delayed orthostatic hypotension, *HC* healthy controls, *normHRBP* normal heart rate and blood pressure response during HUT, *POTS* postural orthostatic tachycardia syndrome, *percent VO*_*2*_ oxygen consumption as percentage of normal values of a population study [19]. The insert figure above right shows the linear regression analysis of HC and norm HRBP patients with a normal CBF (OI-) reduction during HUT: see Additional file 1

Figure 1 shows the regression analysis of the relation between the %peak VO_2_ during CPET and the CBF change during HUT in all three groups. In none of the three groups did the slope of the regression line differ from zero, indicating absence of relationship between exercise performance/deconditioning and orthostatic intolerance. Moreover, the slope of the regression lines did not differ between the three groups.

Among ME/CFS patients, Table 4 shows the distribution of deconditioning as determined with the CPET (no deconditioning = peak VO_2_ ≥ 85%, mild deconditioning = peak VO_2_ 65–85%, and severe deconditioning = peak VO_2_ < 65%) [6] across the different hemodynamic responses during HUT. No significant differences in distribution were found (p = 0.32).

**Table 4:** Distribution of the degree of deconditioning according Parsaik and colleagues across ME/CFS hemodynamic groups (normal HR/BP response, POTS, and dOH), all with a significant CBF decrease as measured during HUT

Degree deconditioning*	NormHRBP (n = 105)(%)	POTS (n = 39) (%)	dOH (n = 27) (%)	Total
No deconditioning: %peak VO_2_ ≥ 85% (n =)	27 (26%)	4 (11%)	7 (26%)	38
Mild deconditioning %peak VO_2_ 65–85% (n =)	33 (31%)	13 (33%)	7 (26%)	53
Severe deconditioning: %peak VO_2_ < 65%(n =)	45 (43%)	22 (56%)	13 (48%)	80
Total	105	39	27	171

Chi square statistics = 5.41(p = 0.25)(3 × 3 table)

*dOH* delayed orthostatic hypotension during HUT, *NormHRBP* normal heart rate and blood pressure response during HUT, *POTS* postural orthostatic tachycardia syndrome during HUT, *%peak VO*_*2*_ oxygen consumption as percentage of normal values of a population study [19]

* Subgrouping of the degree of deconditioning according to Parsaik et al. [6]

**Fig. 2:**
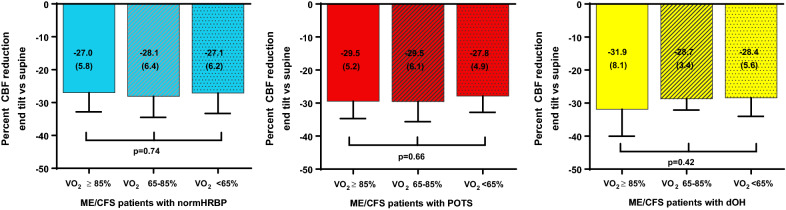
Percent CBF reduction at end-tilt compared to supine and the different degrees of deconditioning, as expressed in the different percentages peak VO_2_, in ME/CFS patients with normHRBP, POTS, and dOH. CBF: cerebral blood flow in ml/min; ME/CFS: myalgic encephalomyelitis/chronic fatigue syndrome patients; NormHRBP: normal heart rate and blood pressure response; dOH: delayed orthostatic hypotension; POTS: postural orthostatic tachycardia syndrome; percent VO_2_: oxygen consumption as percentage of normal values of a population study [19]

Figure 2 displays the degree of CBF reduction in the three ME/CFS patient groups when subdivided according to the degree of deconditioning. In none of the three patient groups was there a significant difference in the degree of CBF reduction across the three categories of deconditioning.

**Fig. 3:**
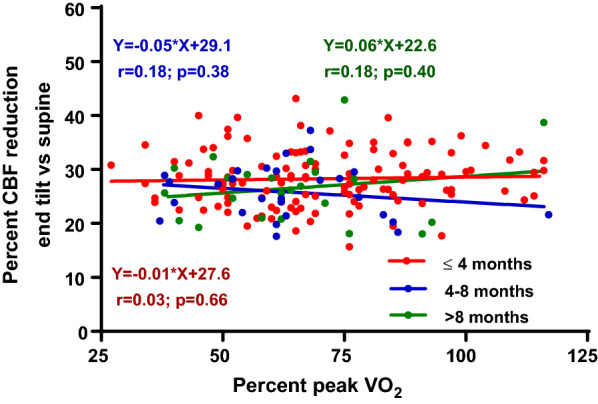
Subgroup analysis of patients with an interval between HUT and CPET of less than 4 months, 4 to 8 months, and 8 to 12 months. CBF: cerebral blood flow in ml/min/kg; percent VO_2_: oxygen consumption as percentage of normal values of a population study [19]

Figure 3 shows the subgroup analysis of patients in whom the interval between HUT and CPET was less than 4 month, in whom the interval was between 4 and 8 month and in whom the interval was between 8 month and 1 year. No difference in the relation between %peak VO_2_ and the CBF reduction was found. None of the three slopes was different from zero. Moreover, the mean %peak VO2 did not differ between groups nor did the percent CBF reduction (data not shown).

## Discussion

In direct contrast to the hypothesis that orthostatic intolerance is caused by deconditioning, this large study in ME/CFS patients identified no relationship between the presence of objectively measured orthostatic intolerance and the degree of objectively measured deconditioning. We examined 199 adults with ME/CFS with a HUT and CPET within a 1-year interval, in whom 86% had an abnormal reduction in CBF during tilt, consistent with orthostatic intolerance. All patients with POTS (n = 39), all with dOH (n = 27), and 105/133 (79%) of those with a normal HR and BP response had a greater than 13% CBF reduction during tilt. In the three groups of patients with an abnormal CBF reduction during HUT there was no relation between the percent peak VO_2_ and the CBF reduction during HUT (see Fig. 1). Also, when categorizing the percent peak VO_2_ as no deconditioning, mild, and severe deconditioning [6], no significant differences were found in the three patient categories based on HUT HR and BP results (see Fig. 2). In the group of ME/CFS patients (n = 28) without the presence of clinical manifestation of OI, the same range in peak VO_2_ results was found (Additional file 1: Fig. S2) in comparison to ME/CFS patients with the presence of clinical manifestation of OI (CBF > 13%). Taken together, these findings provide no support for the hypothesis that deconditioning is a determining factor in the pathogenesis of orthostatic intolerance in ME/CFS.

### Deconditioning

Studies on deconditioning in ME/CFS are conflicting, with some studies suggesting evidence for physical deconditioning [40–43], whereas others found deconditioning to be an inadequate explanation for the exercise intolerance in ME/CFS [44–48].

In general, the term deconditioning is widely used but less well defined in many studies. Deconditioning can be defined as reversible changes/loss of function in body systems due to physical inactivity. Any organ or system can undergo deconditioning: the cardiovascular system, muscles, bones, lungs, the digestive system, the urinary system, blood, the endocrine system, the skin, and the nervous system. Decline in muscle strength and muscle bulk is the most important and consistently reported feature of deconditioning. Reduced peak VO_2_ and decreased cardiac output during exercise are also linked to deconditioning but may primarily result from the reduction in muscle bulk. Muscle strength has been studied In ME/CFS patients using the hand grip strength test, showing reduced hand grip strength compared to healthy controls and MS patients [49]. Nevertheless, measuring exercise capacity/peak VO2 is considered to be the gold standard for assessing deconditioning [6]. In a recent meta-analysis Franklin and coworkers reviewed the available literature on peak VO_2_ in ME/CFS patients versus HC [50]. The authors concluded that despite substantial between-study variability, the available evidence indicates that ME/CFS patients have a significantly lower peak VO_2_ compared to HC. One recent study found in ME/CFS patients a positive correlation between the hand grip strength and peak VO_2_ [51]. The causes of a reduced exercise performance/peak VO_2_ in ME/CFS patients involve both skeletal muscle fatigue and an altered central nervous system innervation (reviewed by Jammes and Retornaz [52]). Moreover, chronotropic incompetence during exercise in ME/CFS patients [53], and cardiovascular deconditioning [41] have also been shown to contribute to the VO_2_ reduction.

The relation between deconditioning and hemodynamic abnormalities obtained by HUT has mainly been emphasized in POTS patients. POTS has been related to deconditioning in a number of studies [6–8, 54], and all these studies advocate exercise training as part of the treatment, implying that deconditioning is at least a contributor to the pathophysiology of POTS. However, alternative explanations for POTS include blood volume reduction [55], mast cell activation disorders [56], peripheral autonomic neuropathy [55, 57–61], high levels of norepinephrine while standing [62], and genetic abnormalities [63]. Importantly, in the present study 11% of the ME/CFS patients with POTS had no signs of deconditioning and 33% had mild deconditioning (see Table 4). Irrespective of the degree of deconditioning, all ME/CFS patients with POTS showed an abnormal CBF reduction during tilt table testing. If there had been a clear relationship between the degree of CBF reduction (objectively confirmed OI) and the degree of %peak VO_2_ reduction (objectively confirmed deconditioning), then this would have provided support for the hypothesis that exercise therapy would be beneficial for treating OI in ME/CFS. Instead, our data suggest instead that exercise therapy alone is unlikely to be effective in improving OI symptoms in this patient population, and that effective treatment of the orthostatic intolerance is more likely to lead to improved function. This holds true not only for ME/CFS patients with POTS, but also for ME/CFS patients with a normal HR and BP response and ME/CFS patients with dOH. As a clinical implication therefore, exercise therapy is not likely to solve the problem of OI, at least in patients with ME/CFS and should therefore be part of a treatment suggestion carefully. Other types of treatment might be more successful in improving patient symptomatology.

Reduced blood volume and abnormal venous pooling are present in POTS and also in other forms of orthostatic intolerance. In an earlier study of ME/CFS patients without POTS we confirmed the relation between blood volume reduction and increase in OI complaints and the relation between blood volume reduction and the reduction in %peak VO_2_ [64, 65]. Furthermore, we have also shown a larger cardiac index reduction during HUT in ME/CFS patients compared to healthy volunteers, supporting the pooling hypothesis [66].

### Limitations

The patients included in this study were a subset of stable ME/CFS patients with a HUT and a CPET within a 1 year interval. Stability of disease was confirmed by review of patient charts by an experienced clinician. Furthermore HUT was performed in patients not on OI medication or using compression stockings. This may have introduced bias. The maximum interval between HUT and CPET was set at 1 year. However, the subgroup analysis of patients with an interval less than 4 month, between 4 and 8 month and between 8 month and 1 year showed no deviation from the main study findings using the entire sample, namely that there was no relation between the percent peak VO2 and CBF reduction. Nevertheless, prospective confirmation of our findings is needed. Our clinic evaluates patients suspected of having ME/CFS. We cannot comment on whether our results in ME/CFS patients with POTS can be extended to those with POTS alone (without ME/CFS)—a group that typically has milder functional impairments—as we included no patients with POTS alone in our study. This question deserves attention in future studies.

## Conclusion

In spite of the commonly held view that there is a causal relation between deconditioning and OI, this study provides no support for this hypothesis. In ME/CFS patients, the objective measure of OI as defined by CBF reduction during HUT was not related to deconditioning as defined by the %peak VO2 obtained during CPET.

## Supplementary Information

**Additional file 1.** **Table S1.** Baseline characteristics of HC and ME/CFS patients with a normal HR and BP response and a normal CBF reduction during HUT; **Table S2.** CPET results of HC and ME/CFS patients with a normal HR and BP response and a normal CBF reduction during HUT; **Table S3.** HUT results in healthy controls and ME/CFS patients with a normal HR and BP response and a normal CBF reduction during HUT; **Figure S1** Flow diagram showing the flow of recruitment and patient flow explaining reasons for exclusion and number of patients analyzed; **Figure S2.** Correlation between the percent CBF reduction at end-tilt compared to supine and the percentage peak VO2 for HC (n=22) and for ME/CFS patients with a normal HR and BP response and a normal CBF reduction during HUT (n=28)

## Data Availability

The raw data supporting the conclusions of this manuscript will be made available by the authors, without undue reservation, to any qualified researcher.
