# Supplementary material for: Deconditioning does not explain orthostatic intolerance in ME/CFS (myalgic encephalomyelitis/chronic fatigue syndrome)
Source: J Transl Med. 2021 May 4;19:193. doi: 10.1186/s12967-021-02819-0 (PMC8097965; doi:10.1186/s12967-021-02819-0)
Supplement: Supplementary file 1 — Additional file 1. Table S1. Baseline characteristics of HC and ME/CFS patients with a normal HR and BP response and a normal CBF reduction during HUT; Table S2. CPET results of HC and ME/CFS patients with a normal HR and BP response and a normal CBF reduction during HUT; Table S3. HUT results in healthy controls and ME/CFS patients with a normal HR and BP response and a normal CBF reduction during HUT; Figure S1 Flow diagram showing the flow of recruitment and patient flow explaining reasons for exclusion and number of patients analyzed; Figure S2. Correlation between the percent CBF reduction at end-tilt compared to supine and the percentage peak VO2 for HC (n=22) and for ME/CFS patients with a normal HR and BP response and a normal CBF reduction during HUT (n=28) [file 12967_2021_2819_MOESM1_ESM.docx]

Additional file

Table S1. Baseline characteristics of HC and ME/CFS patients with a normal HR and BP response and a normal CBF reduction during HUT

|  | Group 1  HC (n=22) | Group 2  OI - (n=28) | p-value (unpaired t-test) |
| --- | --- | --- | --- |
| Male/female | 7/15 | 12/16 | Chi-square test: p=0.42 |
| Age (years) | 46 (11) | 44 (10) | p=0.46 |
| Height (cm) | 174 (9) | 175 (11) | p=0.96 |
| Weight (kg) | 75 (13) | 78 (18) | p=0.42 |
| BMI (kg/m^2^) | 24.5 (3.7) | 25.7 (4.8) | p=0.37 |
| BSA (m^2^) | 1.89 (0.20) | 1.93 (0.25) | p=0.56 |
| Dis duration (years)* | NA | 12 (9.3-20) |  |

*Median with IQR

Table S2. CPET results of HC and ME/CFS patients with a normal HR and BP response and a normal CBF reduction during HUT

| CPET data | Group 1  HC (n=22) | Group 2  OI - (n=28) | p-value (unpaired t-test) |
| --- | --- | --- | --- |
| HR rest (bpm) | 69 (11) | 89 (17) | p<0.0001 |
| HR peak (bpm) | 145 (19) | 155 (19) | p=0.08 |
| SBP rest (mmHg) | 124 (10) | 127 (20) | p=0.71 |
| DBP rest (mmHg) | 76 (5) | 87 (8) | p=0.005 |
| SBP peak (mmHg) | 175 (10) | 168 (21) | p=0.41 |
| DBP peak (mmHg) | 95 (14) | 100 (5) | p=0.49 |
| VT VO_2_ (ml/min/kg) | 16 (4) | 13 (4) | p=0.008 |
| Peak VO_2_ (ml/min/kg) | 27 (5) | 23 (8) | p=0.08 |
| %VT VO_2_ | 56 (12) | 44 (15) | p=0.003; |
| %peak VO_2_ | 93 (16) | 79 (22) | p=0.015 |
| RER | 1.05 (0.11) | 1.08 (0.09) | p=0.27 |

Table S3. HUT results in healthy controls and ME/CFS patients with a normal HR and BP response and a normal CBF reduction during HUT

| HUT data | Group 1  HC (n=22) | Group 2  OI - (n=28) | p-value (unpaired t-test) |
| --- | --- | --- | --- |
| HR supine (bpm) | 63 (12) | 69 (8) | p=0.06 |
| HR end tilt (bpm) | 78 (16) | 84 (12) | p=0.13 |
| SBP supine (mmHg) | 128 (26) | 137 (15) | p=0.12 |
| SBP end tilt (mmHg) | 125 (16) | 136 (18) | p=0.04 |
| DBP supine (mmHg) | 81 (7) | 79 (6) | p=0.26 |
| DBP end tilt (mmHg) | 81 (8) | 86 (8) | p=0.04 |
| CBF supine (ml/min) | 629 (96) | 616 (106) | p=0.66 |
| CBF end tilt (ml/min) | 596 (97) | 586 (102) | p=0.72 |
| %change CBF (%) | -5.3 (2.6) | -5.0 (2.3) | p=0.58 |

Figure S1 Flow diagram showing the flow of recruitment and patient flow explaining reasons for exclusion and number of patients analyzed.

Legend: CBF: cerebral blood flow; CPET: cardiopulmonary exercise testing; HUT: head-up tilt testing; ME/CFS: myalgic encephalomyelitis/chronic fatigue syndrome; OI: orthostatic intolerance.

Figure S2. Correlation between the percent CBF reduction at end-tilt compared to supine and the percentage peak VO_2_ for HC (n=22) and for ME/CFS patients with a normal HR and BP response and a normal CBF reduction during HUT (n=28)

Legend BP: blood pressure; CBF: cerebral blood flow; HC: healthy controls; HR: heart rate; HUT: head-up tilt testing; ME/CFS: myalgic encephalomyelitis/chronic fatigue syndrome; VO_2_: oxygen consumption on cardiopulmonary exercise testing
